# Aqueous Self-Assembly of Block Copolymers to Form Manganese Oxide-Based Polymeric Vesicles for Tumor Microenvironment-Activated Drug Delivery

**DOI:** 10.1007/s40820-020-00447-9

**Published:** 2020-06-11

**Authors:** Yalei Miao, Yudian Qiu, Mengna Zhang, Ke Yan, Panke Zhang, Siyu Lu, Zhongyi Liu, Xiaojing Shi, Xubo Zhao

**Affiliations:** Green Catalysis Center, College of Chemistry, and Laboratory Animal Center, Zhengzhou University, Zhengzhou, 450001 People’s Republic of China

**Keywords:** Polymer, Aqueous self-assembly, Vesicles, Tumor microenvironment, Drug delivery system

## Abstract

**Highlights:**

- The formation of manganese oxide induces self-assembly of block copolymers to form polymeric vesicles.
- The polymeric vesicles possessed strong stability and high drug loading capacity.
- The drug-loaded polymeric vesicles have been demonstrated, especially in in vivo studies, to exhibit a higher efficacy of tumor suppression without known cardiotoxicity.

**Abstract:**

Molecular self-assembly is crucially fundamental to nature. However, the aqueous self-assembly of polymers is still a challenge. To achieve self-assembly of block copolymers [(polyacrylic acid–*block*–polyethylene glycol–*block*–polyacrylic acid (PAA_68_–*b*–PEG_86_–*b*–PAA_68_)] in an aqueous phase, manganese oxide (MnO_2_) is first generated to drive phase separation of the PAA block to form the PAA_68_–*b*–PEG_86_–*b*–PAA_68_/MnO_2_ polymeric assembly that exhibits a stable structure in a physiological medium. The polymeric assembly exhibits vesicular morphology with a diameter of approximately 30 nm and high doxorubicin (DOX) loading capacity of approximately 94%. The transformation from MnO_2_ to Mn^2+^ caused by endogenous glutathione (GSH) facilitates the disassembly of PAA_68_–*b*–PEG_86_–*b*–PAA_68_/MnO_2_ to enable its drug delivery at the tumor sites. The toxicity of DOX-loaded PAA_68_–*b*–PEG_86_–*b*–PAA_68_/MnO_2_ to tumor cells has been verified in vitro and in vivo. Notably, drug-loaded polymeric vesicles have been demonstrated, especially in in vivo studies, to overcome the cardiotoxicity of DOX. We expect this work to encourage the potential application of polymer self-assembly.
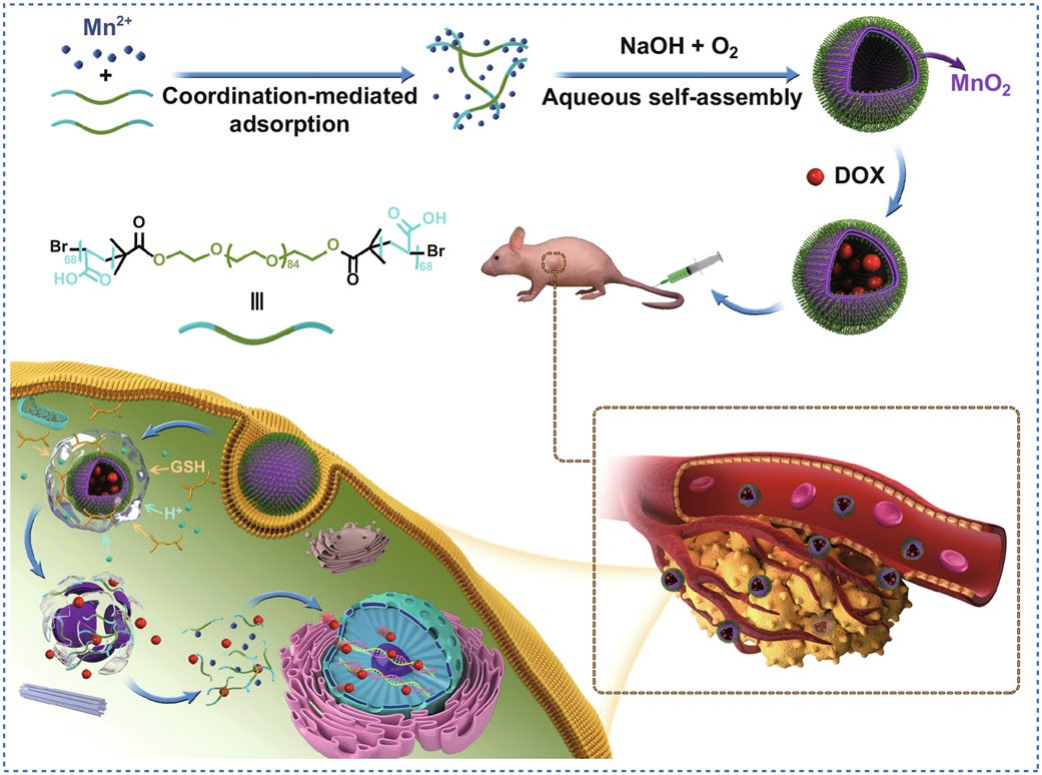

**Electronic supplementary material:**

The online version of this article (10.1007/s40820-020-00447-9) contains supplementary material, which is available to authorized users.

## Introduction

In nature, self-assembly is one of the most crucial approaches that enables the building of micro- and nanostructures [1]. In biology, from structures of plant to those of multicellular organisms, it plays an important role in generating sophisticated superstructures through different interactions between a large number of building blocks [2–4]. Inspired by nature, polymer self-assembly has attracted extensive attention since the 1960s and serves as a “bottom-up” strategy for building complexes naturally [5, 6]. The self-assembly of block copolymers in solution developed by Armes, Kataoka, Discher, and Eisenberg has been utilized to design a series of polymeric architectures [7–10].

In principle, block copolymers can be self-assembled into a wide variety of polymer architectures, but in practice this is usually achieved in co-solvents, often with the aid of both water and an organic solvent [8–10]. As far as we know, self-assembly in nature occurs in aqueous phases, not organic phases, and aqueous self-assembly of block copolymer is an environmentally friendly method with the absence of organic solvents or surfactants. It is important to note that the use of organic solvents or surfactants in traditional self-assembly methods has restricted their application because of the side effects to humans. No matter the application, polymeric architectures formed by block copolymer self-assembly in drug delivery have already become an intriguing focus for a number of research areas, especially in cancer treatments.

The advancement of high-quality therapeutic methods based on polymeric architectures has attracted considerable attention for the treatment of cancer over the past 10 years [11, 12]. Moreover, we have gradually increased our understanding of tumor microenvironments and cells that contain various cooperating components (such as lysosomes and mitochondria) [13–15]. This understanding has facilitated the advancement of numerous nanoparticle-based drug delivery systems (nano-DDSs), both inorganic and organic, to fight against cancers [11, 16, 17]. Organic nanoparticle-based DDSs, especially the polymer micelle-based DDSs, are considered a major strategy to alleviate the side effects of antitumor drugs and improve their therapeutic efficacy [18, 19]. A wide range of self-assembly methods have been extensively utilized to fabricate polymeric nanoparticles such as polymerization- or precipitation-induced self-assembly [20, 21].

However, the inherently inferior stability of polymer micelle-based DDSs at low concentrations could be harmful to humans or impede high-quality therapy [22]. Both covalent cross-linking bonds (including disulfide, borate ester, Schiff base, and ketal bonds) and non-covalent interactions (including electrostatic, hydrophobic, and coordination interaction) have been widely used to develop nano-DDSs for overcoming this limitation [23–29]. However, only a few of these stable inorganic cross-linked block copolymer architectures with a redox potential-responsive property can be utilized to deliver anticancer agents by responding to tumor microenvironments. Therefore, researchers are enthusiastically developing stable block copolymer micelles that can fulfill their target delivery [30, 31].

Although numerous advancements have been achieved, there are still some major barriers that need to be overcome, such as the need for enhanced stability to prevent premature leakage of anticancer agents during the delivery process, selective delivery at tumor sites, and biodegradability of polymeric micelle-based DDSs after completion of drug delivery. However, no easy-to-use fabrication system has been developed. Transformation of MnO_2_ to Mn^2+^ ions has attracted extensive attention because of the stability of MnO_2_ in biological fluids and its disintegration by endogenous glutathione (GSH) inside lysosomes and endosomes at tumor sites [32, 33].

Moreover, Mn^2+^ ions released from the MnO_2_ component within MnO_2_-based systems can be easily metabolized by the kidneys [34]. Therefore, the MnO_2_ component was introduced in the fabrication of biomaterials because of its remarkable biocompatibility [35]. Few reported studies have described the in situ self-assembly of block copolymers using MnO_2_ formation in an aqueous phase. Based on the current knowledge of self-assembly of block copolymers, we hypothesized that block copolymer self-assembly is achievable by MnO_2_ formation in an aqueous phase without additional agents such as organic solvents or surfactants to form a stable polymeric architecture, which could extend the capability of self-assemblies beyond traditional methods.

**Scheme 1:**
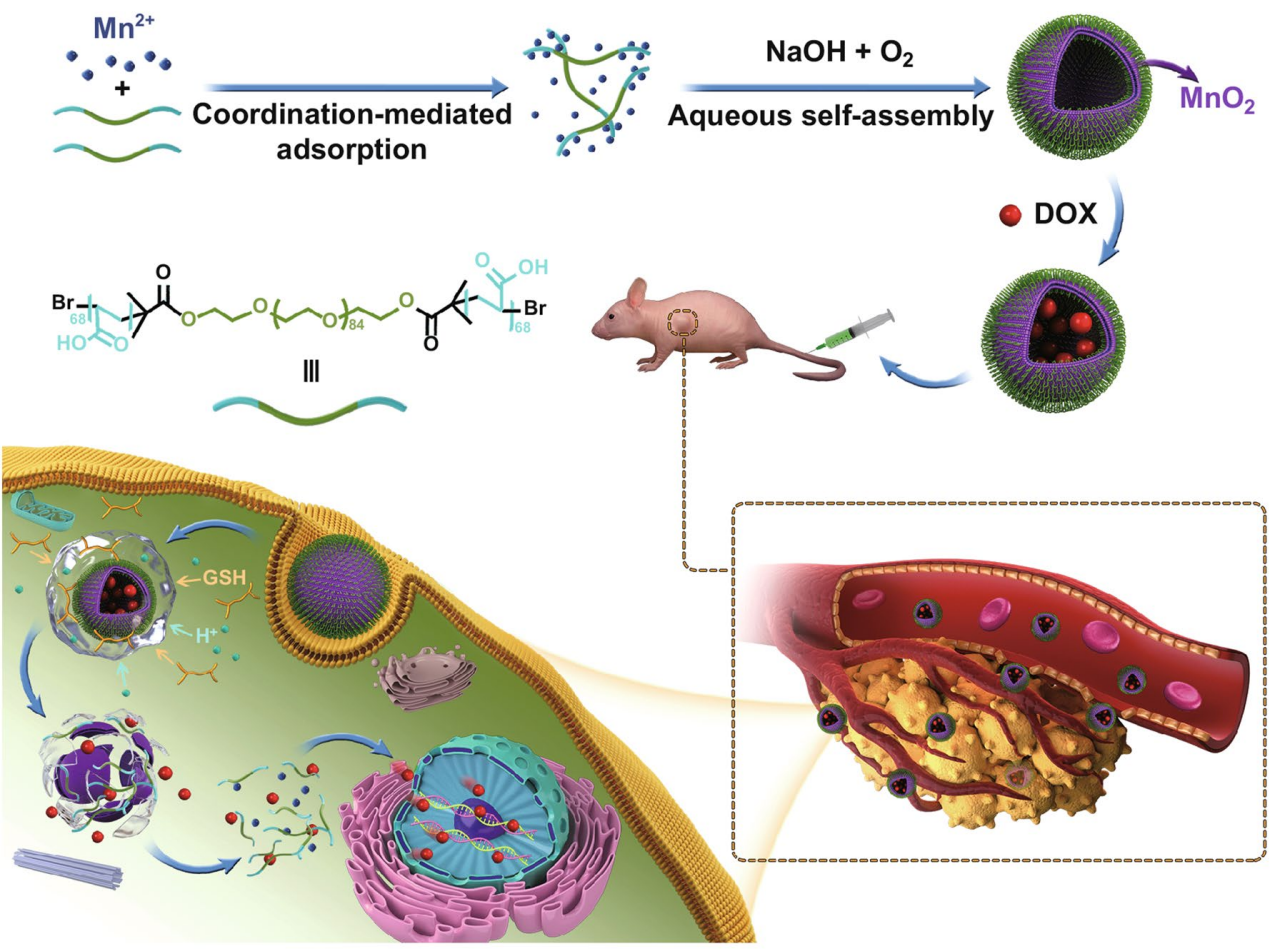
Schematic illustration of aqueous self-assembly of polymer and its tumor microenvironment-activated release

Herein, we describe the use of the self-assembly approach in an aqueous phase in the fabrication of polymeric vesicles with high stability for selective delivery of anticancer agents. The formation of MnO_2_ under mild conditions is used to induce the in situ self-assembly of block copolymers (PAA_68_–*b*–PEG_86_–*b*–PAA_68_) to form PAA_68_–*b*–PEG_86_–*b*–PAA_68_/MnO_2_ polymeric vesicles (Scheme 1), which exhibit the advantage of stability, which prevents the diffusion of encapsulated anticancer agents during the delivery process.

Thus, for the PAA_68_–*b*–PEG_86_–*b*–PAA_68_/MnO_2_ vesicles, MnO_2_ not only serves as a nucleating agent, but it also acts as an interlocking agent in adjusting the self-assembly process of block copolymer with the goal of optimizing their morphology and structural stability. In this design, the introduction of MnO_2_ enhances the structural stability of these PAA_68_–*b*–PEG_86_–*b*–PAA_68_/MnO_2_ vesicles. Compared to the silica/organosilica cross-linked block copolymer architectures [22], the PAA_68_–*b*–PEG_86_–*b*–PAA_68_/MnO_2_ vesicles can respond to GSH and weak acidic conditions to unload their cargos.

Moreover, these polymeric vesicles were further degraded to hydrophilic linear polymers and Mn^2+^ ions after effectively delivery (Scheme 1). On the other hand, the as-released Mn^2+^ ions from the decomposition of the PAA_68_–*b*–PEG_86_–*b*–PAA_68_/MnO_2_ vesicles as a T_2_ contrast agent could endow the DDSs with excellent characteristics for magnetic resonance imaging (MRI) [36]. Therefore, through the self-assembly of copolymers by the formation of MnO_2_ in an aqueous phase, polymeric micelles with controlled morphology and stability were fabricated for the selective delivery of anticancer agents, which may be extended to the development of polymeric architectures.

## Experimental Section

### Materials

Polyethylene glycol (HO–PEG_86_–OH) and doxorubicin (DOX) hydrochloride were obtained from Beijing Kaizheng Bioengineering Development Co. Ltd. *Tert*-butyl acrylate (*t*BA) was obtained from J & K Chem. Ltd. Copper (I) bromide [Cu(I)Br, 99.5%], *N,N,N*′*,N*′*,N*″-pentamethyl diethylenetriamine (PMDETA, 99% purity), dimethylformamide (DMF), 2-bromoisobutyryl bromide (BIBB, 98% purity), sodium hydroxide (NaOH), and manganese chloride tetrahydrate (MnCl_2_·4H_2_O) were obtained from Aladdin Ind. Co. Tetrahydrofuran (THF) and toluene were provided by Tianjin Chem. Co. Ltd. Fetal bovine serum (FBS) was purchased from ScienCell (Carlsbad, CA, USA).

Leibovitz’s L15 medium, 4′,6-diamidino-2-phenylindole (DAPI), paraformaldehyde, and cell counting kit-8 (CCK-8) were obtained from Boster Biotechnology (Wuhan, China). Dulbecco’s modified Eagle’s medium (DMEM) was obtained from Thermo Scientific. The human breast cancer MCF-7 (Catalog No.: SCSP-531) and human embryonic kidney HEK-293 (Catalog No.: GNHu43) cell lines were obtained from the Cell Bank of the Chinese Academy of Sciences (Shanghai, China). The two cell lines were used within 20 passages in this study. Additionally, deionized water was adopted for all experiments.

### Synthesis of PAA_68_–*b*–PEG_86_–*b*–PAA_68_

The synthetic steps of these copolymers are shown in the Supporting Information according to our previously reported studies [18, 19].

### Aqueous Self-assembly of PAA_68_–*b*–PEG_86_–*b*–PAA_68_

The formation of MnO_2_ was used to induce self-assembly of block copolymers (PAA_68_–*b*–PEG_86_–*b*–PAA_68_) to form the MnO_2_–polymer hybrid vesicles (PAA_68_–*b*–PEG_86_–*b*–PAA_68_/MnO_2_). Briefly, 50 mg PAA_68_–*b*–PEG_86_–*b*–PAA_68_ was dispersed in 50 mL water under magnetic stirring with slight ultrasound sonication for 10 min using the Ultrasonic Cleaner (KQ-300DE). Then, 10 mL MnCl_2_ solution (360 mg) was slowly added with vigorous magnetic stirring. The mixture was transferred into dialysis tubes (molecular weight cutoff [MWCO], 1000) and then immersed in abundant deionized water to remove excess Mn^2+^ ions.

After dialysis against deionized water, the pH value of the mixture was adjusted to pH 11 using a 1.0 M NaOH aqueous solution. The PAA_68_–*b*–PEG_86_–*b*–PAA_68_/MnO_2_ vesicles were obtained under vigorous stirring for 24 h and stored at 4 °C. To optimize the effect of the Mn^2+^ concentrations on the morphology of the MnO_2_–polymer hybrids, 60 and 180 mg MnCl_2_·4H_2_O were successively added to induce the self-assembly of PAA_68_–*b*–PEG_86_–*b*–PAA_68_ according to the above procedures.

### DOX Loading

DOX-loaded PAA_68_–*b*–PEG_86_–*b*–PAA_68_/MnO_2_ was prepared using 10 mg DOX and 10 mg PAA_68_–*b*–PEG_86_–*b*–PAA_68_/MnO_2_ in 10 mL phosphate-buffered saline (PBS) solution (pH 7.4) according to the reported procedures [18]. After centrifugation, the DOX-loaded PAA_68_–*b*–PEG_86_–*b*–PAA_68_/MnO_2_ was obtained as a precipitate and the DOX mass in the supernatant was determined using an ultraviolet (UV) spectrophotometer at 482 nm. The DOX encapsulation efficiency and DOX loading capacity for PAA_68_–*b*–PEG_86_–*b*–PAA_68_/MnO_2_ were evaluated using the following equations: DOX loading capacity (%) = (DOX mass in polymeric vesicle/polymeric vesicle mass) × 100 and DOX encapsulation efficiency (%) = (DOX mass in polymeric vesicle/feeding DOX mass) × 100.

Additionally, the above measurements were repeated with three samples from different batches to obtain the final loading capacity and encapsulation efficiency of DOX. The effect of two different DOX concentrations (2.5 mg DOX vs 10.0 mg PAA_68_–*b*–PEG_86_–*b*–PAA_68_/MnO_2_ and 5.0 mg DOX vs 10.0 mg PAA_68_–*b*–PEG_86_–*b*–PAA_68_/MnO_2_) on the loading capacity, drug release, and average hydrodynamic diameters (*D*_h_) of DOX-loaded PAA_68_–*b*–PEG_86_–*b*–PAA_68_/MnO_2_ were studied according to the above procedures and were, respectively, denoted as DOX_1_-loaded PAA_68_–*b*–PEG_86_–*b*–PAA_68_/MnO_2_ and DOX_2_-loaded PAA_68_–*b*–PEG_86_–*b*–PAA_68_/MnO_2_.

### Cell Toxicity Assays

The 3-(4,5-dimethylthiazol-2-yl)-2,5-diphenyltetrazolium bromide (MTT) assay was performed to evaluate the biocompatibility of PAA_68_–*b*–PEG_86_–*b*–PAA_68_/MnO_2_ vesicles with HEK-293 and MCF-7 cells. Furthermore, the growth inhibitory ability of DOX-loaded PAA_68_–*b*–PEG_86_–*b*–PAA_68_/MnO_2_ was also evaluated on MCF-7 cells using the MTT assay. After the cells were seeded in 96-well plates at 5000 cells/well for 24 h and had reached a confluency of over 80%, 200 μL medium containing the PAA_68_–*b*–PEG_86_–*b*–PAA_68_/MnO_2_, DOX-loaded PAA_68_–*b*–PEG_86_–*b*–PAA_68_/MnO_2_, or free DOX samples of different concentrations were added and incubated at 37 °C with 0.5% CO_2_ for 1, 2, or 3 days.

Typically, the concentrations of PAA_68_–*b*–PEG_86_–*b*–PAA_68_/MnO_2_, free DOX, or DOX-loaded PAA_68_–*b*–PEG_86_–*b*–PAA_68_/MnO_2_ (equivalent DOX concentrations) were 0.1, 0.5, 1.0, 2.5, and 5.0 μg mL^−1^. Then, the cell viability was detected using the MTT assay: 20 μL MTT (5 mg mL^−1^) was added to each well and incubated for 4 h. Then, the cell-bound dye was dissolved in 150 μL dimethyl sulfoxide (DMSO) and the absorbance was recorded using a microplate reader at 490 nm.

### Controlled Release

For controlled release evaluation of DOX-loaded PAA_68_–*b*–PEG_86_–*b*–PAA_68_/MnO_2_, 50 mg was equally dispersed in samples of 10.0 mL PBS (pH 7.4, pH 7.4 in the presence of 10 μM GSH, pH 6.5, pH 5.0, and pH 5.0 in the presence of 10 mM GSH). After filtration using 0.2-μm ultrafiltration membranes, the PBS dispersions of DOX-loaded PAA_68_–*b*–PEG_86_–*b*–PAA_68_/MnO_2_ were transferred into dialysis tubes (MWCO, 1000) and immersed in 140.0 mL of PBS at the corresponding pH at 37 °C. A 5.0 mL aliquot of the solution was collected to detect the drug concentration using a UV spectrophotometer at 482 nm at specific time intervals (0, 1, 3, 6, 12, 24, 48, and 60 h), and 5.0 mL fresh PBS of the corresponding pH was supplemented after each sampling.

Each controlled release analysis was repeated three times, and the final cumulative release ratio of DOX was the average of three measurements. DOX release from both DOX_1_-loaded PAA_68_–*b*–PEG_86_–*b*–PAA_68_/MnO_2_ and DOX_2_-loaded PAA_68_–*b*–PEG_86_–*b*–PAA_68_/MnO_2_ was analyzed at pH 7.4 in the presence of 10 μM GSH at pH 5.0 and 10 mM GSH according to the above procedures.

### Confocal Laser Scanning Microscopy Analysis

The confocal laser scanning microscopy (CLSM) technique was used to investigate the cellular uptake of DOX-loaded PAA_68_–*b*–PEG_86_–*b*–PAA_68_/MnO_2_ vesicles and free DOX by MCF-7 cells at excitation wavelengths of 482 nm for DOX and 406 nm for Hoechst, as reported previously [36].

### Flow Cytometric Analysis

Flow cytometry was used to detect the intracellular release of the as-loaded DOX from DOX-loaded PAA_68_–*b*–PEG_86_–*b*–PAA_68_/MnO_2_ vesicles by measuring the cell-associated fluorescence using a flow cytometer (BD FACSCalibur) at specific time intervals (1, 3, 6, 9, and 12 h), after free DOX (2.5 μg mL^−1^) or DOX-loaded PAA_68_–*b*–PEG_86_–*b*–PAA_68_/MnO_2_ (equivalent DOX concentration: 2.5 μg mL^−1^) was added to the MCF-7 cells and incubated at 37 °C exposed to an atmosphere of 0.5% CO_2_. The experimental details are available from our previously reported studies [37].

### Animal Experiments

Female nude BALB/c mice aged 5–6 weeks were obtained from Hunan Slack Scene of Laboratory Animal Co., Ltd. (Hunan, China) and Shanghai Laboratory Animal Center (Shanghai, China). Animals were treated according to protocols established by the ethics committee of Zhengzhou University, and the in vivo experiments were approved and conducted in accordance with the guidelines of the ethics committee of Zhengzhou University. MCF-7 cells (1 × 10^6^) were subcutaneously injected into the right flank of the nude mice (*n* = 5 per group).

When the tumor volumes reached 100 mm^3^, the mice were randomized into saline, PAA_68_–*b*–PEG_86_–*b*–PAA_68_/MnO_2_, free DOX, and DOX-loaded PAA_68_–*b*–PEG_86_–*b*–PAA_68_/MnO_2_ groups. The groups were treated with corresponding samples intravenously (5 mg kg^−1^ day^−1^) once every 3 days. Tumor size was then measured every 3 days using Vernier calipers. Tumor volume was calculated using the following formula: volume = (length × width^2^)/2. Tumors were weighed after 31 days, and, subsequently, the hearts and livers were sectioned for histological evaluation using hematoxylin and eosin (H&E) staining.

### Characterization

Proton (^1^H) nuclear magnetic resonance (NMR) spectra were recorded using a Bruker Avance (II) 400 MHz spectrometer at room temperature. Automatic tuning module (ATM) and double probe were utilized for the NMR spectrometer. ^1^H NMR spectra were measured in deuterated chloroform (CDCl_3_) or deuterium oxide (D_2_O, heavy water) using tetramethylsilane as the internal standard, and the concentration of the samples was 2 mg mL^−1^. Fourier transform infrared (FT-IR) spectra were recorded using a Bruker IFS 66 v/s IR spectrometer at 4000–400 cm^−1^ with a resolution of 4 cm^−1^. The number average molecular weight (*M*_n_) and polydispersity (PDI) of the copolymers were measured using gel permeation chromatography (GPC) in THF at 35 °C. The vesicular morphology was analyzed using a JEM-1200 EX/S transmission electron microscope (TEM).

X-ray photoelectron spectroscopy (XPS) of PAA_68_–*b*–PEG_86_–*b*–PAA_68_/MnO_2_ was performed using an Elementar Vario EL instrument (Elementar Analysensysteme GmbH, Munich, Germany). An Agilent 7700× inductively coupled plasma mass spectrometer (ICP-MS) was used to detect the content of MnO_2_ in the PAA_68_–*b*–PEG_86_–*b*–PAA_68_/MnO_2_ vesicles. For ICP-MS analysis, the samples were treated with 10% hydrochloric acid for 8 h and then heated to remove the hydrochloric acid. The dynamic light scattering (DLS) measurements were performed using a light scattering system BI-200SM device. The release performance of DOX-loaded PAA_68_–*b*–PEG_86_–*b*–PAA_68_/MnO_2_ was assessed using a PerkinElmer Lambda 35 UV–Vis spectrometer at room temperature.

## Results and Discussion

### Preparation of PAA_68_–*b*–PEG_86_–*b*–PAA_68_

**Fig. 1:**
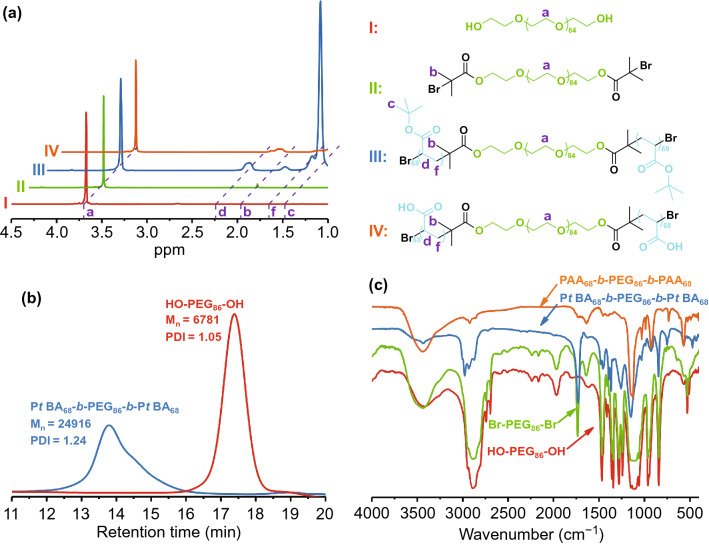
**a** ^1^H NMR spectra of HO–PEG_86_–OH (I), Br–PEG_86_–Br (II), P*t*BA_68_–*b*–PEG_86_–*b*–P*t*BA_68_ (III), and PAA_68_–*b*–PEG_86_–*b*–PAA_68_ (IV). **b** GPC spectra of HO–PEG_86_–OH and P*t*BA_68_–*b*–PEG_86_–*b*–P*t*BA_68_. **c** FT-IR spectra of HO–PEG_86_–OH, Br–PEG_86_–Br, P*t*BA_68_–*b*–PEG_86_–*b*–P*t*BA_68_, and PAA_68_–*b*–PEG_86_–*b*–PAA_68_

To achieve aqueous self-assembly, a water-soluble block copolymer PAA_68_–*b*–PEG_86_–*b*–PAA_68_ was prepared using rational design. Furthermore, we selected biocompatible PEG as a unique block to surmount the biological barriers. In this work, the PAA_68_–*b*–PEG_86_–*b*–PAA_68_ triblock copolymer was synthesized using a combination of acylation reaction, atomic transfer radical polymerization (ATRP), and hydrolysis reaction (Fig. S1). In the ^1^H NMR spectrum of HO–PEG_86_–OH, the characteristic peak at 3.68 ppm was assigned to the inner methylene protons (Fig. 1a). After the acylation reaction, the presence of the –CH_3_ proton peak at 1.94 ppm in the ^1^H NMR spectrum of Br–PEG_86_–Br (Fig. 1a) clearly confirmed the successful acylation reaction.

In addition to the characteristic proton peaks of the inner methylene and methyl protons, the characteristic proton peaks at 1.44 ppm clearly revealed the presence of *t*-butyl groups in P*t*BA–*b*–PEG_86_–*b*–P*t*BA (Fig. 1a). The peak area ratio of a to c was used to evaluate the polymerization degree to *t*BA, and the well-defined P*t*BA_68_–*b*–PEG_86_–*b*–P*t*BA_68_ block copolymer was successfully obtained. According to the gel permeation chromatography (GPC) results (Fig. 1b), the M_n_ of P*t*BA_68_–*b*–PEG_86_–*b*–P*t*BA_68_ was 24,916 Da, which was in good agreement with the ^1^H NMR spectral result of P*t*BA_68_–*b*–PEG_86_–*b*–P*t*BA_68_. Moreover, the PDI (M_w_/M_n_) of PtBA_68_–*b*–PEG_86_–*b*–P*t*BA_68_ was 1.24, indicating the existence of a narrow MW distribution. Figure 1c shows that the characteristic absorbance peaks at 1735 and 1394 cm^−1^ were associated with ester groups of Br–PEG_86_–Br and *t*-butyl groups, respectively, to P*t*BA–*b*–PEG_86_–*b*–P*t*BA. These findings were consistent with the results of the ^1^H NMR spectral analysis. The characteristic proton peaks of the *t*-butyl groups disappeared in the ^1^H NMR spectrum of PAA_68_–*b*–PEG_86_–*b*–PAA_68_, demonstrating the successful hydrolysis of *t*-butyl groups in P*t*BA_68_–*b*–PEG_86_–*b*–P*t*BA_68_. In addition, the characteristic absorbance of carboxyl stretching vibration in *t*-butyl groups at 1735 cm^−1^ disappeared in the FT-IR spectrum of the PAA_68_–*b*–PEG_86_–*b*–PAA_68_, demonstrating its generation from P*t*BA_68_–*b*–PEG_86_–*b*–P*t*BA_68_. Therefore, the resultant PAA_68_–*b*–PEG_86_–*b*–PAA_68_ copolymer possessed abundant carboxyl groups to chelate the Mn^2+^ ions [36, 38].

### Preparation of PAA_68_–*b*–PEG_86_–*b*–PAA_68_/MnO_2_

**Fig. 2:**
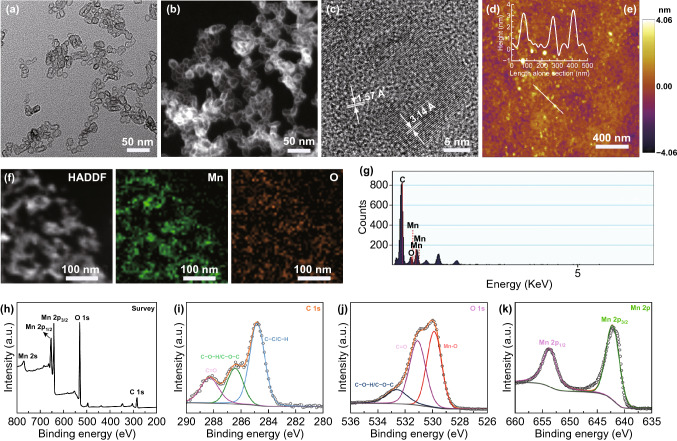
**a** TEM, **b** and **f** HADDF-STEM, **c** high-magnification TEM, **d** and **e** AFM height images of PAA_68_–*b*–PEG_86_–*b*–PAA_68_/MnO_2_. **g** EDS, **h** XPS, and high-resolution (HR) XPS spectra of **i** C 1*s*, **j** O 1*s*, and **k** Mn 2*p* of PAA_68_–*b*–PEG_86_–*b*–PAA_68_/MnO_2_ are presented

The formation of MnO_2_ was incorporated to induce in situ self-assembly of the water-soluble block copolymer in an aqueous phase. Inspired by the diversities of the polymer topologies, the MnO_2_–polymer architectures with vesicular morphology were obtained through phase separation. After adequately mixing the PAA_68_–*b*–PEG_86_–*b*–PAA_68_ and excess MnCl_2_·4H_2_O in 60 mL aqueous solution under magnetic stirring, 1 M NaOH aqueous solution was introduced into the above mixtures. Subsequently, the MnO_2_ formed induced in situ self-assembly of PAA_68_–*b*–PEG_86_–*b*–PAA_68_ to obtain the PAA_68_–*b*–PEG_86_–*b*–PAA_68_/MnO_2_ architectures. The morphology of the MnO_2_–polymer hybrids changed from irregular spheres to uniform vesicles as the Mn^2+^ feed concentrations increased as shown in Figs. 2a and S2.

We made an important discovery that well-defined assemblies of PAA_68_–*b*–PEG_86_–*b*–PAA_68_ were not obtained with lower amounts of MnCl_2_·4H_2_O (1 and 3 mg mL^−1^), but amounts higher than 6 mg mL^−1^ produced well-defined vesicles. TEM images (Fig. 2a) showed that PAA_68_–*b*–PEG_86_–*b*–PAA_68_/MnO_2_ exhibited a vesicular morphology with a diameter of approximately 30 nm, which was consistent with the results of the determinations using the high-angle angular dark-field scanning transmission electron microscopy (HADDF-STEM, Fig. 2b) and atomic force microscopy (AFM, Fig. 2d, e). Moreover, the HADDF-STEM image of PAA_68_–*b*–PEG_86_–*b*–PAA_68_/MnO_2_ also revealed the vesicular morphology.

**Fig. 3:**
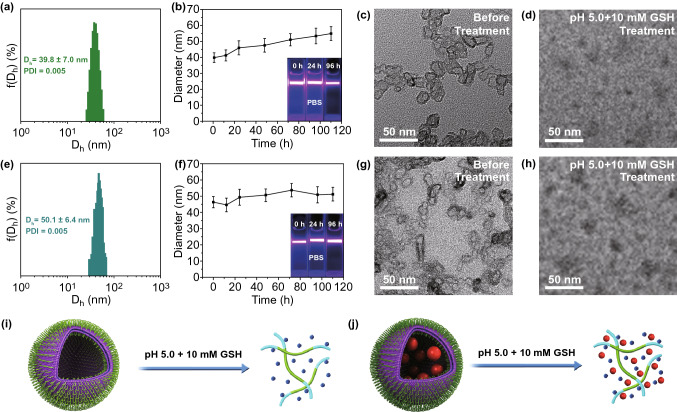
**a** *D*_h_ distributions, **b** hydrodynamic particle size and Tyndall effect of dispersed stability of PAA_68_–*b*–PEG_86_–*b*–PAA_68_/MnO_2_ in PBS at different times. TEM images **c** before and **d** after dissociation of PAA_68_–*b*–PEG_86_–*b*–PAA_68_/MnO_2_. **e** *D*_h_ distributions, **f** hydrodynamic particle size and Tyndall effect of dispersed stability of DOX-loaded PAA_68_–*b*–PEG_86_–*b*–PAA_68_/MnO_2_ in PBS at different times. TEM images **g** before and **h** after dissociation of DOX-loaded PAA_68_–*b*–PEG_86_–*b*–PAA_68_/MnO_2_. Schematic illustrations of **i** disintegration of PAA_68_–*b*–PEG_86_–*b*–PAA_68_/MnO_2_ and **j** DOX-loaded PAA_68_–*b*–PEG_86_–*b*–PAA_68_/MnO_2_ at pH 5.0 with 10 mM GSH

Notably, the AFM result showed that PAA_68_–*b*–PEG_86_–*b*–PAA_68_/MnO_2_ displayed a closed form, similar to vesicles with a hollow structure. Especially in the DLS (Fig. 3a), importantly, the hydrodynamic diameter of the PAA_68_–*b*–PEG_86_–*b*–PAA_68_/MnO_2_ architecture was much bigger than the size observed with TEM. This finding was attributed to the extreme stretch of hydrophilic PEG segments in the PAA_68_–*b*–PEG_86_–*b*–PAA_68_/MnO_2_ architecture in the aqueous phase, whereas these vesicles collapsed during the TEM analysis.

As shown in Fig. 2f, the oxygen and Mn were uniformly distributed in the polymeric vesicles, indicating that Mn^2+^ ions were transformed into MnO_2_. Moreover, the result agreed with the energy-dispersive spectrometer (EDS) analysis (Fig. 2g) and XPS results (Fig. 2h), further indicating the existence of MnO_2_. To confirm the chemical valence of elemental Mn, the high-resolution (HR) XPS spectra of PAA_68_–*b*–PEG_86_–*b*–PAA_68_/MnO_2_ were analyzed and are presented in Fig. 2k. As shown in Fig. 2i-k, the C 1 s spectra of these samples were deconvoluted into three peaks that were assigned to a C–H/C–C band at 284.8 eV, C–O–C band at 286.7 eV, and C=O band at 288.2 eV, indicating the existence of a copolymer.

The deconvolutions of the O 1*s* spectra of PAA_68_–*b*–PEG_86_–*b*–PAA_68_/MnO_2_ indicated the obvious formation of MnO_2_ in these polymeric architectures because of the special peaks at 530.6 eV. In the Mn 2*p* spectrum (Fig. 2k), two specific peaks at binding energies of 653.1 and 641.1 eV were observed, indicating the existence of Mn^4+^ in MnO_2_ [39]. Additionally, the HRTEM image of the PAA_68_–*b*–PEG_86_–*b*–PAA_68_/MnO_2_ vesicle (Fig. 2c) exhibited characteristic lattice fringes with interplanar distance of 1.57 and 3.14 Å, also demonstrating the presence of MnO_2_ [40]. Moreover, the ICP-MS used to detect the content of MnO_2_ in the PAA_68_–*b*–PEG_86_–*b*–PAA_68_/MnO_2_ vesicles revealed that it was approximately 4 wt%.

Based on these results, polymeric vesicles with a controlled morphology and stability were successfully fabricated using the self-assembly approach in an aqueous phase. Compared to polymerization-induced self-assembly [41], MnO_2_ formation gradually made the soluble PAA block insoluble, which drove in situ self-assembly to form the MnO_2_–polymer polymeric vesicles. During the self-assembly, formation of MnO_2_ was used to drive the phase separation of one of the blocks and further induce in situ self-assembly of block copolymers (PAA_68_–*b*–PEG_86_–*b*–PAA_68_) to form the MnO_2_–polymer hybrids (PAA_68_–*b*–PEG_86_–*b*–PAA_68_/MnO_2_). Consequently, we determined that the self-assembly process in water should be performed via three steps: (1) complete dissolution of the copolymer segments in the aqueous phase at the initial stage, (2) reduction in the solvability of the PAA/MnO_2_ segments to drive the phase separation in the MnO_2_ formation process, and (3) nucleation of the PAA/MnO_2_ segments to induce the self-assembly of the copolymer chains at the final stage. Because of the vesicular morphology of PAA_68_–*b*–PEG_86_–*b*–PAA_68_/MnO_2_, we further explored its functions and properties for broader applications.

### Structural Stability and Degradability of PAA_68_–*b*–PEG_86_–*b*–PAA_68_/MnO_2_

After successfully fabricating the polymer–inorganic architecture, we tested the structural stability of the PAA_68_–*b*–PEG_86_–*b*–PAA_68_/MnO_2_ vesicles in PBS. Figure 3b shows that PAA_68_–*b*–PEG_86_–*b*–PAA_68_/MnO_2_ exhibited a series of stable hydrodynamic diameters during 110 h. In addition, the Tyndall effects, which depend on the support of the corresponding dispersion system at different points in time, were observed as shown in Fig. 3b, demonstrating the excellent stability of the PAA_68_–*b*–PEG_86_–*b*–PAA_68_/MnO_2_ structure [42]. Subsequently, the dispersion time was extended to 20 days in PBS, and the corresponding Tyndall effect (Fig. S3) further indicated that the PAA_68_–*b*–PEG_86_–*b*–PAA_68_/MnO_2_ vesicles possessed excellent dispersity.

When the dispersion time was extended to 100 days in PBS, stable dispersion was maintained as shown in Fig. S5b. We also explored the structural stability of the PAA_68_–*b*–PEG_86_–*b*–PAA_68_/MnO_2_ vesicles in DMEM with FBS (10%, v/v) as shown in Fig. S4. When the PAA_68_–*b*–PEG_86_–*b*–PAA_68_/MnO_2_ hybrid vesicles were treated with DMEM with FBS (10%, v/v), an obvious Tyndall effect was observed even when the reaction time was extended to 6 days, indicating their favorable stability. We believed that the favorable stability of the PAA_68_–*b*–PEG_86_–*b*–PAA_68_/MnO_2_ vesicles was due to the introduction of MnO_2_.

Therefore, the introduction of MnO_2_ played a crucial role in the preparation of PAA_68_–*b*–PEG_86_–*b*–PAA_68_/MnO_2_ vesicles that exhibited excellent dispersity and good stability. As expected, the complete hydrolysis preserved abundant carboxyl groups and further supported the MnO_2_ cross-linking to introduce pH/GSH dual sensitivity in the final product. After treatment with an acidic and reductive dual-sensitive trigger (pH 5.0 and 10 mM GSH), the dissociation of PAA_68_–*b*–PEG_86_–*b*–PAA_68_/MnO_2_ was observed as presented in Figs. 3d and S5c compared to that of fresh PAA_68_–*b*–PEG_86_–*b*–PAA_68_/MnO_2_ (Figs. 3c and S5a), indicating the disintegration of PAA_68_–*b*–PEG_86_–*b*–PAA_68_/MnO_2_.

These degradable products, which have received US Food and Drug Administration (FDA) approval, included PEG and PAA [43, 44]. Other products include the as-released Mn^2+^ ions that can be metabolized easier by the kidneys [35, 36]. The transformation of MnO_2_ to Mn^2+^ ions could contribute to the disintegration of PAA_68_–*b*–PEG_86_–*b*–PAA_68_/MnO_2_ (Fig. 3i), which would facilitate the wider application of polymer–inorganic hybrids as DDS, especially in cancer treatments where the delivery of anticancer agents is the focus.

### Dual-Responsive Drug Release from DOX-Loaded PAA_68_–*b*–PEG_86_–*b*–PAA_68_/MnO_2_ In Vitro

Taking advantage of the electrostatic interaction in an aqueous phase [45], the chemotherapeutic agent (DOX), a broad spectrum anticancer drug with known cardiotoxicity [46], was encapsulated in PAA_68_–*b*–PEG_86_–*b*–PAA_68_/MnO_2_. The PAA_68_–*b*–PEG_86_–*b*–PAA_68_/MnO_2_ polymeric vesicles showed a high drug encapsulation efficiency and drug loading capacity (both up to ~ 94%) derived from their abundant carboxyl groups. Moreover, the interpenetrating network between the copolymer and MnO_2_ in PAA_68_–*b*–PEG_86_–*b*–PAA_68_/MnO_2_ polymeric vesicles also probably contributed to the efficient loading of DOX.

After DOX loading, the vesicular morphology of DOX-loaded PAA_68_–*b*–PEG_86_–*b*–PAA_68_/MnO_2_ vesicles was observed (Fig. 3g) and its *D*_h_ (Fig. 3e) increased more than that of the PAA_68_–*b*–PEG_86_–*b*–PAA_68_/MnO_2_ vesicles (Fig. 3a), suggesting the introduction of DOX molecules. The structural stability of DOX-loaded PAA_68_–*b*–PEG_86_–*b*–PAA_68_/MnO_2_ was evaluated as shown in Fig. 3f, implying that DOX-loaded PAA_68_–*b*–PEG_86_–*b*–PAA_68_/MnO_2_ exhibited a favorable stability in PBS during 110 h. In addition, the Tyndall effect, which depends on the support of the corresponding dispersion system at different points in time, was observed as shown in Fig. 3f, demonstrating the excellent stability of DOX-loaded PAA_68_–*b*–PEG_86_–*b*–PAA_68_/MnO_2_.

**Fig. 4:**
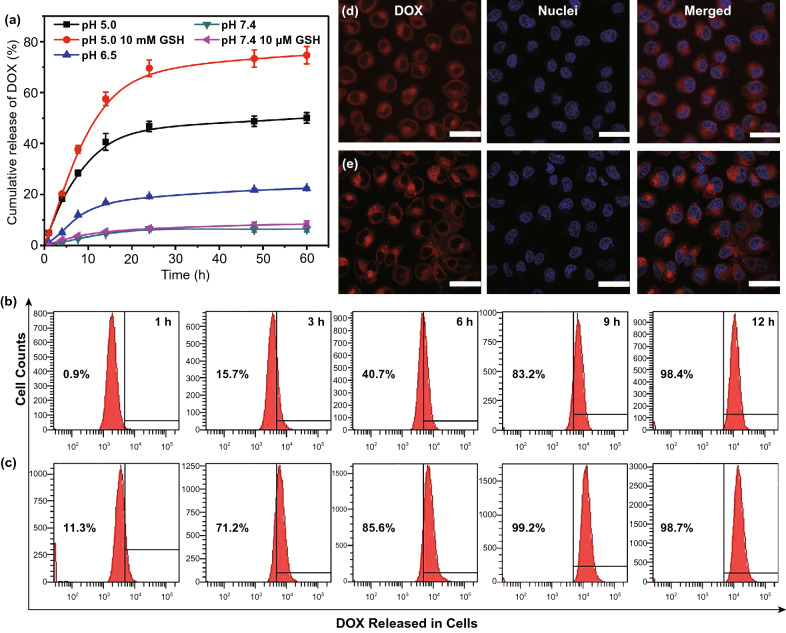
**a** Cumulative release of DOX from DOX-loaded PAA_68_–*b*–PEG_86_–*b*–PAA_68_/MnO_2_ in simulated body fluids. **b** Intracellular DOX release from DOX-loaded PAA_68_–*b*–PEG_86_–*b*–PAA_68_/MnO_2_ in MCF-7 cells over a range of different times. **c** Free DOX used as control group. **d** CLSM visualization of cellular DOX release from DOX-loaded PAA_68_–*b*–PEG_86_–*b*–PAA_68_/MnO_2_ after incubation for 9 h. **e** Free DOX used as control group, scale bar: 50 μm. For CLSM and flow cytometric analyses, equivalent DOX dose was 2.5 μg mL^−1^

After treatment with an acidic and reductive dual-sensitive trigger (pH 5.0 and 10 mM GSH), the dissociation of DOX-loaded PAA_68_–*b*–PEG_86_–*b*–PAA_68_/MnO_2_ was observed as shown in Fig. 3h, j, indicating the disintegration of DOX-loaded PAA_68_–*b*–PEG_86_–*b*–PAA_68_/MnO_2_. The DOX release curve showed that only 6% drug cumulative release occurred under normal conditions, whereas the drug cumulative release proportion reached 22% and 50% with decreasing pH from 6.5 to 5.0 within 60 h (Fig. 4a). In addition, the introduction of 10 μM GSH only slightly increased DOX release compared to that at pH 7.4 as presented in Fig. 4a.

After treatment with GSH as high as 10 mM (a biologically relevant level [2-10 mM]) at pH 5.0 [18, 36], the DOX release was obviously facilitated, and a passive release plateau was observed over 24 h. Importantly, the final cumulative release ratio of DOX culminated at 75% at pH 5.0 in the presence of 10 mM GSH as presented in Fig. 4a. When the feed concentrations of DOX were decreased from 1.00 to 0.25 or 0.50 mg mL^−1^, a similar drug encapsulation efficiency of 95% or 93% was obtained. After DOX loading, the DOX_1_-loaded PAA_68_–*b*–PEG_86_–*b*–PAA_68_/MnO_2_ and DOX_2_-loaded PAA_68_–*b*–PEG_86_–*b*–PAA_68_/MnO_2_ vesicles displayed a similar *D*_h_ of approximately 50 nm as shown in Fig. S6.

Furthermore, the DOX_1_-loaded PAA_68_–*b*–PEG_86_–*b*–PAA_68_/MnO_2_ and DOX_2_-loaded PAA_68_–*b*–PEG_86_–*b*–PAA_68_/MnO_2_ vesicles also presented a similar trend of DOX release (Fig. S6) to that of DOX-loaded PAA_68_–*b*–PEG_86_–*b*–PAA_68_/MnO_2_ (Fig. 4a) under the same conditions, indicating that the introduction of DOX molecules had a negligible influence on the structure of PAA_68_–*b*–PEG_86_–*b*–PAA_68_/MnO_2_. The intracellular DOX release from DOX-loaded PAA_68_–*b*–PEG_86_–*b*–PAA_68_/MnO_2_ was precisely evaluated at different time intervals as shown in Fig. 4b. At each incubation time, the DOX-loaded PAA_68_–*b*–PEG_86_–*b*–PAA_68_/MnO_2_ exhibited a slow cellular release in contrast to that of free DOX as shown in Fig. 4c, indicating that the DOX-loaded DDS was first degraded before and then the drugs were unloaded.

The DOX-loaded PAA_68_–*b*–PEG_86_–*b*–PAA_68_/MnO_2_ unloaded almost all its cargos when the incubation time was increased to 12 h, which almost agreed with the accumulative release of DOX from DOX-loaded PAA_68_–*b*–PEG_86_–*b*–PAA_68_/MnO_2_ at pH 5.0 in the presence of 10 mM GSH as presented in Fig. 4a. The CLSM technique was used to detect the intracellular distribution of the as-released DOX molecules from DOX-loaded PAA_68_–*b*–PEG_86_–*b*–PAA_68_/MnO_2_ as visualized in MCF-7 cells. After incubation for 9 h, the obvious red fluorescence of DOX molecules was observed at the cell nucleus as shown in Fig. 4d, suggesting that the DOX molecule was more efficiently delivered to the cell nucleus by the PAA_68_–*b*–PEG_86_–*b*–PAA_68_/MnO_2_ than the free DOX was (Fig. 4e). These results demonstrate that the polymeric vesicles responded to tumor acidity and reduction-triggered activation, achieving on-demand release of DOX.

### Cytotoxicity of DOX-Loaded PAA_68_–*b*–PEG_86_–*b*–PAA_68_/MnO_2_ In Vitro

To further elucidate the toxicity of DOX-loaded PAA_68_–*b*–PEG_86_–*b*–PAA_68_/MnO_2_, its effects on the viability of MCF-7 cells were evaluated and free DOX and PAA_68_–*b*–PEG_86_–*b*–PAA_68_/MnO_2_ were selected as two controls as shown in Fig. S7. After treatment with PAA_68_–*b*–PEG_86_–*b*–PAA_68_/MnO_2_, both MCF-7 and HEK-293 cells maintained a viability of approximately 90% over the full tested concentration range over 3 days, indicating that PAA_68_–*b*–PEG_86_–*b*–PAA_68_/MnO_2_ exhibited a favorable biocompatibility. After incubation with DOX-loaded PAA_68_–*b*–PEG_86_–*b*–PAA_68_/MnO_2_, the viability of MCF-7 cells decreased from 92.3 to 54.2% as the DOX equivalent dose to DOX-loaded PAA_68_–*b*–PEG_86_–*b*–PAA_68_/MnO_2_ increased from 0.1 to 5.0 μg within 1 day.

More intriguingly, the inhibitory effects of DOX-loaded PAA_68_–*b*–PEG_86_–*b*–PAA_68_/MnO_2_ on MCF-7 cells were apparently enhanced when the incubation time was increased to 2 or 3 days. In addition, DOX-loaded PAA_68_–*b*–PEG_86_–*b*–PAA_68_/MnO_2_ displayed a similar trend with that of free DOX against MCF-7 cells (Fig. S7). These analyses validate the important fact that DOX-loaded PAA_68_–*b*–PEG_86_–*b*–PAA_68_/MnO_2_ induced cell death, whereas the PAA_68_–*b*–PEG_86_–*b*–PAA_68_/MnO_2_ vesicles exhibited favorable biocompatibility.

### Therapeutic Effect of DOX-Loaded PAA_68_–*b*–PEG_86_–*b*–PAA_68_/MnO_2_ In Vivo

**Fig. 5:**
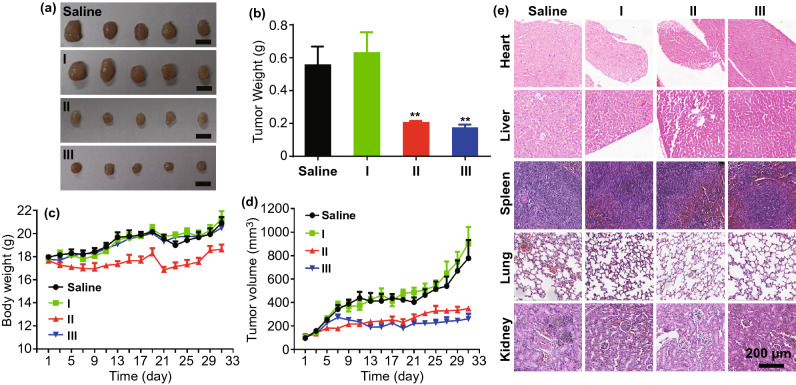
Cancer chemotherapy in vivo using PAA_68_–*b*–PEG_86_–*b*–PAA_68_/MnO_2_ (I), free DOX (II), and DOX-loaded PAA_68_–*b*–PEG_86_–*b*–PAA_68_/MnO_2_ (III) in MCF-7 tumor-bearing mice. **a** Digital images of tumors from MCF-7 tumor-bearing mice after 31 days in vivo cancer chemotherapy, scale bar: 1 cm. **b** Weight of tumor excised from MCF-7 tumor-bearing mice after treatment. **c** Body weight changes of MCF-7 tumor-bearing mice during therapy. **d** Tumor size change of MCF-7 tumor-bearing mice during therapy. **e** Histological analyses of major tissues from MCF-7 tumor-bearing mice after 31 days in vivo cancer chemotherapy, scale bar: 200 μm

The antitumor efficacy of DOX-loaded PAA_68_–*b*–PEG_86_–*b*–PAA_68_/MnO_2_ in vivo was evaluated in mice bearing MCF-7 orthotopic xenografts. After 31 days, the tumor size apparently increased in the saline group (Fig. 5a). Moreover, the tumor size also showed similar trends in the PAA_68_–*b*–PEG_86_–*b*–PAA_68_/MnO_2_ group. After treatment with DOX-loaded PAA_68_–*b*–PEG_86_–*b*–PAA_68_/MnO_2_ for 31 days, there was an obvious decrease in the tumor size compared to that of both the saline and PAA_68_–*b*–PEG_86_–*b*–PAA_68_/MnO_2_ groups (Fig. 5a). This decrease was consistent with the results of the free DOX group, indicating that DOX-loaded PAA_68_–*b*–PEG_86_–*b*–PAA_68_/MnO_2_ exhibited an excellent antitumor efficacy.

In addition, the tumor tissues of these mice were excised and weighed at the end of the experiment as shown in Fig. 5b, and the results indicate that DOX-loaded PAA_68_–*b*–PEG_86_–*b*–PAA_68_/MnO_2_ group exhibited similar tumor suppression to that in the DOX group. During treatment, the body weight of mice in the DOX-loaded PAA_68_–*b*–PEG_86_–*b*–PAA_68_/MnO_2_ group exhibited a similar trend with that of the PAA_68_–*b*–PEG_86_–*b*–PAA_68_/MnO_2_ group (Fig. 5c). In contrast, after 31 days, the body weight of mice in the free DOX group was obviously reduced by approximately 24%, revealing the severe toxicity of free DOX (Fig. 5c). As shown in Fig. 5d, the tumor size of the DOX-loaded PAA_68_–*b*–PEG_86_–*b*–PAA_68_/MnO_2_ and free DOX group slightly increase from ~ 100 to ~ 220 and 290 mm^3^, respectively.

In contrast, the tumor size of the PAA_68_–*b*–PEG_86_–*b*–PAA_68_ group obviously increases from approximately 100 to approximately 910 mm^3^. According to the histological analyses of the major organs, DOX-loaded PAA_68_–*b*–PEG_86_–*b*–PAA_68_/MnO_2_ did not induce obvious pathological changes in these tissues in contrast to the obvious pathological changes induced by the free DOX group as shown in Fig. 5e. Particularly, the liver and heart of the DOX-loaded PAA_68_–*b*–PEG_86_–*b*–PAA_68_/MnO_2_ group over came the cardiotoxicity of DOX. All the observations demonstrated that DOX-loaded PAA_68_–*b*–PEG_86_–*b*–PAA_68_/MnO_2_ exhibited higher tumor suppression without known cardiotoxicity, most probably because of the unique structure of the MnO_2_–polymer hybrid vesicles.

## Conclusions

In conclusion, a simple and efficient aqueous self-assembly strategy was investigated for the construction of polymeric vesicles. In this self-assembly process, the formation of MnO_2_ not only induced in situ self-assembly of block copolymers, but also served as an interlocking agent to endow the polymeric vesicles with a fairly stable structure for the delivery process. Accordingly, the process of self-assembly is performed in three steps: (1) complete dissolution of the copolymer segments in an aqueous phase at the initial stage, (2) reduction in the solvability of the PAA block to drive the phase separation in the nucleation process, and (3) nucleation of PAA/MnO_2_ segments to induce self-assembly of the copolymer chains at the final stage.

Compared to other polymeric architectures formed using traditional self-assembly strategies, the polymeric vesicles respond to GSH and weak acidic conditions at tumor sites to unloading the cargo and are further degraded to hydrophilic linear polymers and Mn^2+^ ions after achieving drug delivery. DOX-loaded PAA_68_–*b*–PEG_86_–*b*–PAA_68_/MnO_2_ vesicles have been demonstrated, especially in in vivo studies, to overcome the cardiotoxicity of DOX. Moreover, other metallic oxides (including zinc oxide and ferroferric oxide) may also be utilized to induce in situ self-assembly of block copolymers using this strategy. Therefore, the as-proposed self-assembly strategy could facilitate the widening of the fabrication of a variety of polymeric architectures, and we expect that some versatile polymeric systems will be crafted using this environmentally friendly approach.

## Electronic supplementary material

Below is the link to the electronic supplementary material.

Supplementary material 1 (DOCX 935 kb)
