# Supplementary material for: Aqueous Self-Assembly of Block Copolymers to Form Manganese Oxide-Based Polymeric Vesicles for Tumor Microenvironment-Activated Drug Delivery
Source: Nanomicro Lett. 2020 Jun 11;12:124. doi: 10.1007/s40820-020-00447-9 (PMC7770723; doi:10.1007/s40820-020-00447-9)
Supplement: Supplementary file 1 — Supplementary material 1 (DOCX 935 kb) [file 40820_2020_447_MOESM1_ESM.docx]

Supporting Information for

**Aqueous Self-Assembly of Block Copolymers to Form Manganese Oxide-Based Polymeric Vesicles for Tumor Microenvironment-Activated Drug Delivery**

Yalei Miao^1^, Yudian Qiu^1^, Mengna Zhang^1^, Ke Yan^1^, Panke Zhang^1^, Siyu Lu^1^, Zhongyi Liu^1^*, Xiaojing Shi^1^*, Xubo Zhao^1^*

^1^Green Catalysis Center, College of Chemistry, and Laboratory Animal Center, Zhengzhou University, Zhengzhou 450001, People’s Republic of China

*Corresponding author. E-mail: xbz2016@zzu.edu.cn (Xubo Zhao); liuzhongyi@zzu.edu.cn (Zhongyi Liu); shixiaojing@zzu.edu.cn (Xiaojing Shi)

**S1 Experimental Section**

**S1.1 Synthesis of Br–PEG_86_–Br**

After 10.0 g of HO–PEG_86_–OH was dissolved in 150 mL of toluene, approximately 40 mL of toluene with traces of water was removed from the mixture by a zeotropic distillation at reduced pressure. Then 2.5 mL of Triethylamine was added into the solution at 0 °C. Subsequently, 2.0 mL of 2-bromoisobutyryl bromide was added dropwise via a constant pressure funnel during 40 min with magnetic stirring, and the reaction was performed with moderate stirring overnight at room temperature. After most toluene was removed at reduced pressure, the product was precipitated in excess cold ether. The precipitate was dried under vacuum, dissolved in 20 mL of pH 8-9 NaHCO_3_ aqueous solution, and extracted with CH_2_Cl_2_. Subsequently, the organic phase was gathered and dried over MgSO_4_. Finally, CH_2_Cl_2_ was removed completely at reduced pressure to obtain the resultant macroinitiator (Br–PEG_86_–Br).

**S1.2 Synthesis of P*t*BA_68_–*b*–PEG_86_–*b*–P*t*BA_68_**

P*t*BA_68_–*b*–PEG_86_–*b*–P*t*BA_68_ was synthesized via the ATRP of *Tert*-butyl acrylate (*t*BA) with the macroinitiator Br–PEG_86_–Br. An amount of 2.080 g (0.5 mM) of Br–PEG_86_–Br was dissolved in 6 mL of anhydrous Tetrahydrofuran (THF). After the mixture was gassed and degassed under N_2_, 0.172g (1.0 mM) of *N,N,N″,N″*-Pentamethyl diethylenetriamine and 17.920 g (140 mM) of *t*BA were charged under degassing by freeze-pump-thaw in a N_2_ atmosphere, followed by adding 0.143 g (0.1 mM) of CuBr and then degassing. Subsequently, ATRP was carried out at 45 °C for 8 h with the conversion of *t*BA of 100% from the information on ^1^H NMR analysis. The copper catalyst in the resultant solution was removed with an alumina column, after dilution with THF. The block copolymer P*t*BA_68_–*b*–PEG_86_–*b*–P*t*BA_68_ was precipitated in cold ether and dried in vacuum overnight at room temperature.

**S1.3 Hydrolysis of P*t*BA_68_–*b*–PEG_86_–*b*–P*t*BA_68_**

The triblock copolymer was dissolved in 25 mL of CH_2_Cl_2_, and 4 mL of TFA was added and stirred at room temperature for 24 h. Most of the CH_2_Cl_2_ and TFA were removed at reduced pressure by a rotary evaporator. The hydrolytic copolymers of PAA_68_–*b*–PEG_86_–*b*–PAA_68_ were obtained by lyophilization for 6 h.

**S2 Supplementary Figures**





**Fig. S1** Synthetic process of the PAA_68_–*b*–PEG_86_–*b*–PAA_68_ copolymer


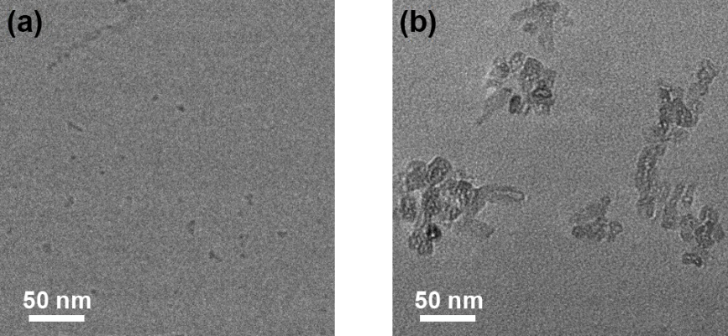


**Fig. S2** TEM images of MnO_2_-polymer hybrids. Images of (**a**) and (**b**) respectively corresponds to the feed concentrations of MnCl_2_•4H_2_O at 1 and 3 mg mL^-1^


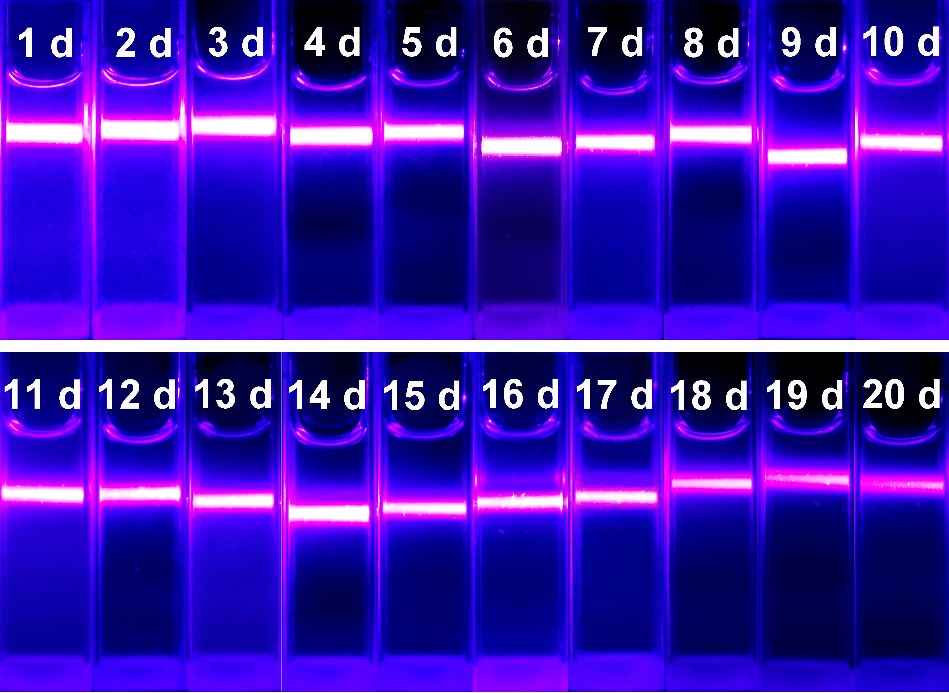


**Fig. S3** Tyndall effects for structural stability of PAA_68_–*b*–PEG_86_–*b*–PAA_68_/MnO_2_ (1 mg mL^-1^) in PBS for 20 days


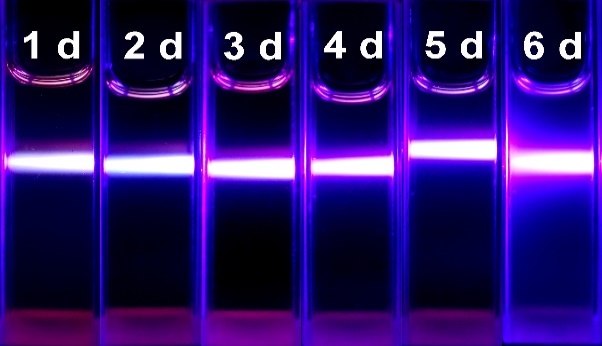


**Fig. S4** Tyndall effects for structural stability of PAA_68_–*b*–PEG_86_–*b*–PAA_68_/MnO_2_ (1 mg mL^-1^) in DMEM with FBS (10%, v/v) for 6 days


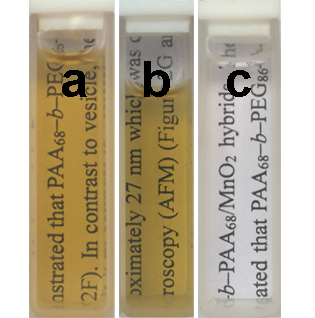


**Fig. S5** Photo images for dispersed stability of PAA_68_–*b*–PEG_86_–*b*–PAA_68_/MnO_2_ in PBS for 1 h (**a**) and 100 days (**b**). Photo image for dissociation of PAA_68_–*b*–PEG_86_–*b*–PAA_68_/MnO_2_ in the presence of 10 mM GSH at pH 5.0 (**c**)

**
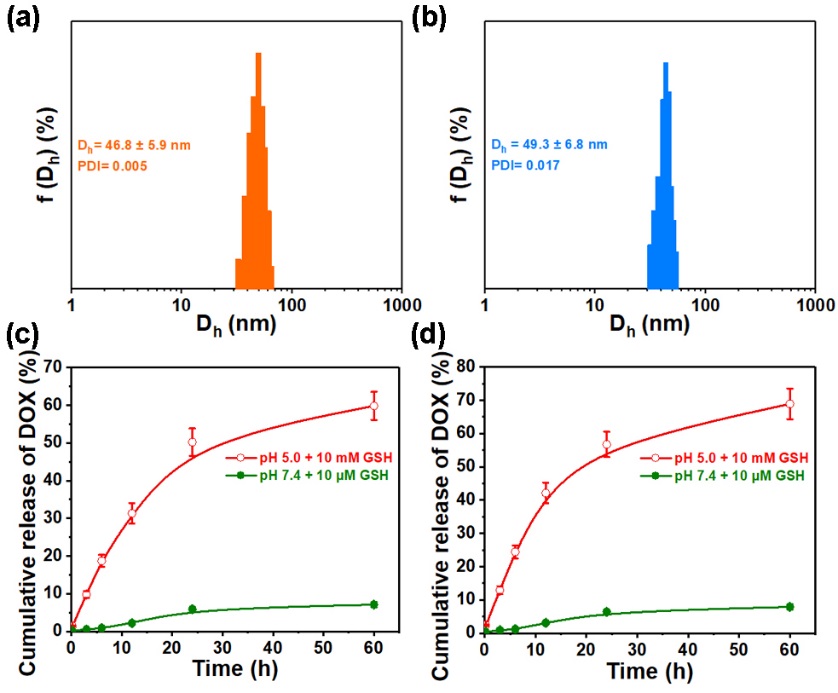
**

**Fig. S6** D_h_ distributions of DOX_1_-loaded PAA_68_–*b*–PEG_86_–*b*–PAA_68_/MnO_2_ (**a**) and DOX_2_-loaded PAA_68_–*b*–PEG_86_–*b*–PAA_68_/MnO_2_ (**b**). The cumulative release of DOX from DOX_1_-loaded PAA_68_–*b*–PEG_86_–*b*–PAA_68_/MnO_2_ (**c**) and DOX_2_-loaded PAA_68_–*b*–PEG_86_–*b*–PAA_68_/MnO_2_ in simulated body fluids (**d**)


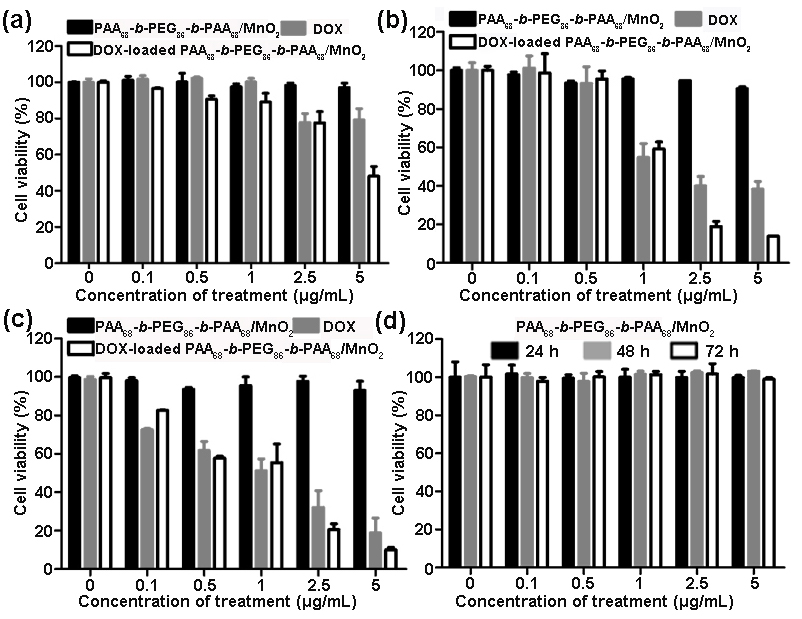


**Fig. S7** Cell viability assay in MCF-7 cells by treatment with PAA_68_–*b*–PEG_86_–*b*–PAA_68_/MnO_2_, free DOX, and DOX-loaded PAA_68_–*b*–PEG_86_–*b*–PAA_68_/MnO_2_ for 24 h (**a**), 48 h (**b**), and 72 h (**c**). Additionally, Cell viability assay in HEK-293 cells by treatment with PAA_68_–*b*–PEG_86_–*b*–PAA_68_/MnO_2_ for different time (**d**)
